# Therapeutic Effects of Electroencephalogram-Based Bioelectric Stimulation on Cognitive–Behavioural Outcomes in Children With Dual Diagnosis of Autism Spectrum Disorder and Intellectual Disability

**DOI:** 10.62641/aep.v53i4.1975

**Published:** 2025-08-05

**Authors:** Jiufang He, Yiping Shi, Xike Wang

**Affiliations:** ^1^Medical School of Guizhou University, 550025 Guiyang, Guizhou, China; ^2^Department of Pediatric Hematology, Zhujiang Hospital of Southern Medical University, 510282 Guangzhou, Guangdong, China; ^3^Department of Pediatric, Guizhou Provincial People's Hospital, 550025 Guiyang, Guizhou, China; ^4^Department of Pediatric, Shanghai Children's Medical Center Guizhou Hospital, 550025 Guiyang, Guizhou, China

**Keywords:** electroencephalogram-based bioelectric stimulation, autism spectrum disorder, intellectual disability, cognitive function, quality of life

## Abstract

**Objectives:**

This investigation evaluates the interventional effects of electroencephalogram-based bioelectric stimulation (EBBS) on intellectual development and behavioural symptoms in children with autism spectrum disorder (ASD) and comorbid intellectual disability (ID).

**Methods:**

By utilising a retrospective cohort design, the research team analysed 310 clinically diagnosed cases of ASD and ID that were stratified into two intervention groups: a conventional group (n = 163) receiving conventional interventions (behavioural applied behaviour analysis (ABA) therapy and structured instruction) and an observation group (n = 147) receiving the same behavioural interventions combined with EBBS. Before and following the treatment, the childhood autism rating scale (CARS), Montreal cognitive assessment (MoCA), developmental age and developmental quotient (DQ) and infants–junior middle school students' social-life abilities scale (S–M) were employed to assess symptom alleviation, cognitive capabilities and quality of life. The levels of serum 25-hydroxyvitamin D [25(OH)D], folic acid (FA) and brain-derived neurotrophic factor (BDNF) were also measured.

**Results:**

After treatment, the observation group showed significantly lower CARS scores; increased post-treatment serum levels of 25(OH)D, FA and BDNF; and improved MoCA scores than the conventional group (*p* < 0.05). Regarding developmental age and DQ, the observation group demonstrated significant improvements in the subscales of fine motor skills, language, adaptive ability and social interaction after intervention (*p* < 0.05). Additionally, the S–M total scores and all quality-of-life indicators were superior in the observation group (*p* < 0.05).

**Conclusion:**

EBBS has the potential to collaboratively enhance the cognitive function, behavioural symptoms and quality of life of children with comorbid ASD and ID.

## Introduction

The global prevalence of autism spectrum disorder (ASD), a complex neurodevelopmental condition marked by social communication impairments, repetitive and stereotypical behaviours and restricted interests, has witnessed a dramatic surge in recent decades. The World Health Organization’s statistics reveal an alarming trend, with a staggering 1 in 54 children globally receiving an ASD diagnosis [1]. The frequent co-occurrence of intellectual disability (ID) is of particular clinical significance, with research suggesting that approximately 30%–50% of individuals with ASD demonstrate comorbid cognitive impairments [2]. This dual diagnosis presents a challenging clinical profile, characterised by profound cognitive impairments, remarkably adaptive functioning deficits and severe learning difficulties, resulting in substantial caregiver burden and socioeconomic effect [3]. Epidemiological studies in China reveal an ASD prevalence of approximately 1%, with ID co-occurring in over 40% of cases. The disorder exhibits a significant male predominance [4]. Although behavioural interventions, rehabilitation training and pharmacological treatments can provide partial symptomatic relief for core ASD features in some ASD cases, their efficacy in enhancing cognitive development among children with comorbid ASD–ID remains suboptimal. This situation underscores the critical need for developing innovative therapeutic strategies to overcome existing treatment limitations [5].

Electroencephalogram (EEG)-based bioelectric stimulation (EBBS) has emerged as a promising non-invasive neuromodulation approach for neurodevelopmental disorders [6]. Through the analysis of individual EEG rhythmic patterns and delivery of biologically inspired electrical signals to targeted brain regions, EBBS can normalise aberrant neural oscillations while enhancing synaptic plasticity and neural circuit reorganisation [7]. Growing evidence suggests EBBS’s therapeutic potential in enhancing attention, executive capabilities and memory across various conditions, from cognitive rehabilitation in post-stroke patients to children with attention deficit hyperactivity disorder and learning difficulties [8, 9]. Nevertheless, its application in ASD–ID therapy remains a largely uncharted territory. A critical gap exists in current ASD research: investigations predominantly emphasise social behaviour modification while largely neglecting dual-dimensional interventions targeting cognitive development and behavioural manifestations [10]. Furthermore, the long-term therapeutic effects of EBBS remain fundamental questions that need to be addressed.

This research concentrates on the impact of EBBS on intellectual growth and behavioural manifestations in children with ASD–ID comorbidity. The imperative nature of this study can be attributed to three key factors. Firstly, the ASD-ID comorbidity presents unique clinical challenges and cognitive and behavioural issues that conventional therapies often fail to address effectively [11]. Secondly, EBBS, through its targeted regulation of abnormal EEG activities, might open a novel avenue of ‘neural remodelling’ for neurodevelopmental disorders [12]. Thirdly, the current literature suffers from methodological limitations, with most studies employing unidimensional outcome measures rather than integrating cognitive metrics [e.g., standardised intelligence quotient (IQ) measures], behavioural assessments (e.g., stereotypic behaviours and emotional dysregulation) and neurophysiological biomarkers [13]. To address these limitations, our study aims to offer evidence-based support for refining the comprehensive intervention strategies for ASD–ID comorbidity, thereby filling a significant void in this research area.

## Materials and Methods

### Study Design

**Fig. 1.**
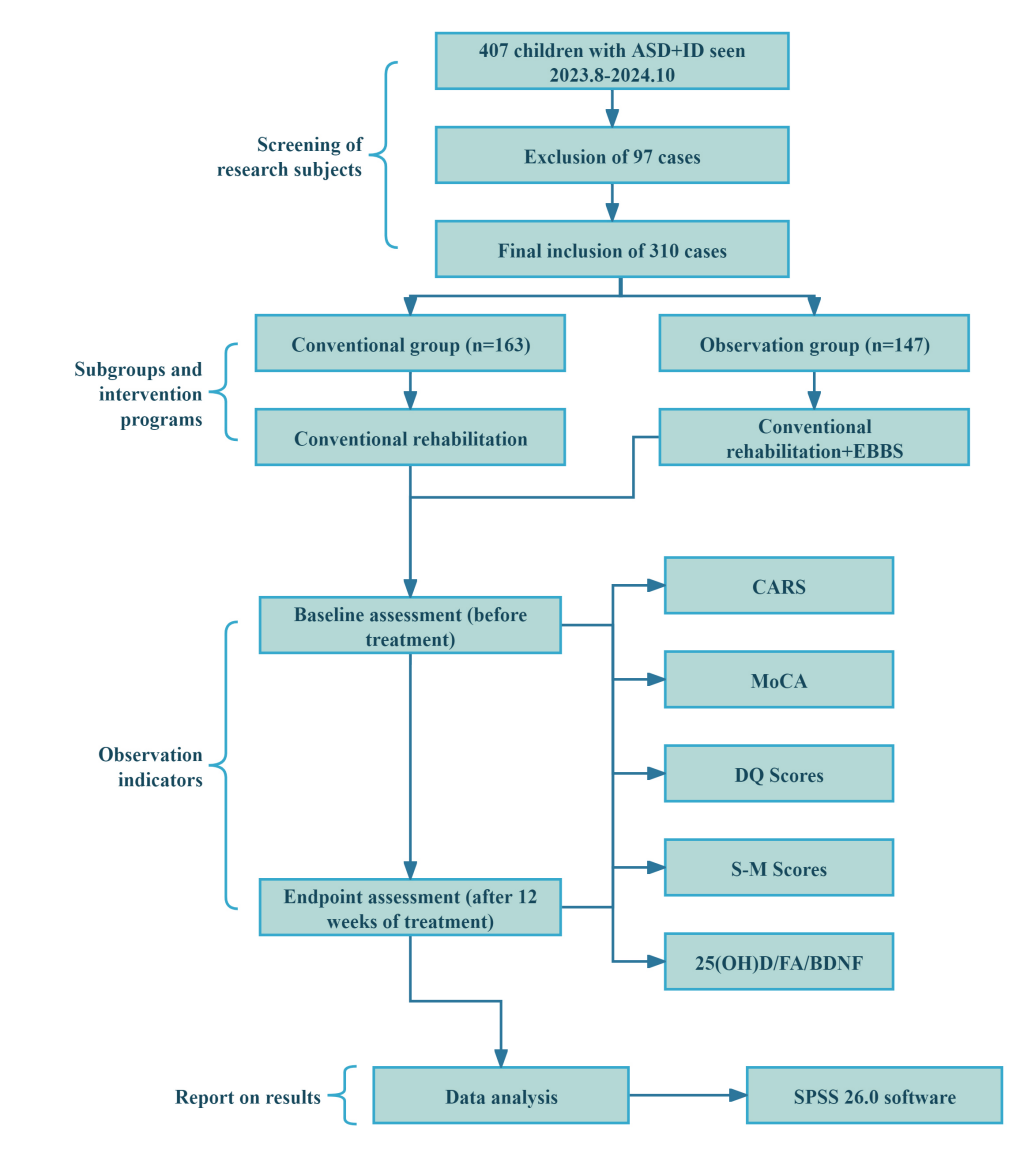
**Flow chart of this study**. Note: EBBS, Electroencephalogram-based bioelectric stimulation; ASD, Autism Spectrum Disorder; CARS, Childhood Autism Rating Scale; 25(OH)D, 25-Hydroxyvitamin D; FA, Folic Acid; BDNF, Brain-Derived Neurotrophic Factor; MoCA, Montreal Cognitive Assessment; DQ, Developmental Quotient; S–M, Infants–Junior Middle School Students’ Social–Life Abilities Scale.

By employing a retrospective approach, this study initially identified 407 paediatric patients diagnosed with ASD–ID who received treatment at our hospital from August 2023 to October 2024. Power analysis performed using G*Power software 3.1 (University of Düsseldorf, Düsseldorf, North Rhine-Westphalia, Germany) (two-tailed test, effect size = 0.3, α = 0.05 and power = 0.95) determined a minimum required sample size of 134 participants. Following the rigorous application of inclusion and exclusion criteria, the final cohort comprised 310 subjects, stratified into two treatment groups: 163 children assigned to conventional therapy (conventional group) and 147 receiving EBBS (observation group). The conventional group received 12 weeks of applied behaviour analysis (ABA) therapy (30 minutes/session, six sessions/week) and structured teaching (30 minutes/session, six sessions/week) with no additional interventions. In addition to the same ABA therapy and structured teaching, the observation group underwent EBBS sessions (20 minutes/day, 5 days/week) using the HB520D system. The main process of this study is shown in Fig. 1. The study protocol received ethical approval from Guizhou Provincial People’s Hospital (Approval Number: 2021-57)’s ethics committee, and all the participants’ legal guardians provided documented informed consent prior to enrolment.

### Inclusion and Exclusion Criteria

The inclusion criteria were as follows: (1) confirmed diagnosis of ASD; (2) concurrent ID as defined in the Diagnostic and Statistical Manual of Mental Disorders [14] and categorised as mild [requiring minimal support (e.g., social communication difficulties with limited impact on daily functioning)], moderate [requiring substantial support (e.g., marked deficits in verbal/nonverbal communication)] and severe [requiring very substantial support (e.g., extreme communication deficits and repetitive behaviours)]; (3) completion of at least 3 months of consistent treatment in our hospital; (4) age <12 years; and (5) availability of complete medical records.

The exclusion criteria were as follows: (1) behavioural presentations not attributable to a primary ASD diagnosis; (2) significant comorbid medical conditions including acute infections, cardiopulmonary dysfunction or severe organ system pathologies; (3) comorbid conditions that could interfere with the treatment of the primary diagnosis; (4) presence of implanted electronic medical devices (e.g., pacemakers); and (5) current or historical diagnosis of seizure disorders.

### Therapeutic Interventions

Conventional rehabilitation protocol: (1) ABA therapy: This evidence-based intervention employs behavioural shaping principles with positive reinforcement to enhance various developmental skills. The structured protocol involves four key phases: therapist instruction delivery, child response elicitation, therapist feedback provision and inter-trial interval. In practice, the therapist designs suitable functional games tailored to the child’s developmental functions and interacts with the child. During this interactive process, the therapist provides prompts or assistance to guide the child in performing correct behaviours. As the training progresses, the amount of assistance is gradually decreased until the child can independently execute the correct actions without any external help. A short interlude is scheduled between the training of every two decomposed actions. This treatment was administered six times per week, with each session lasting 30 minutes. (2) Structured teaching: This method focuses on targeted training to address deficits in language, communication, sensory perception and behavioural issues. By using clear and easily understandable visual cues, the therapist tailors the training content and requirements to the child’s abilities and elaborates them to the child in detail. The training scope covers a wide range of aspects, including fine motor skills, gross motor imitation, cognitive abilities, perception skills, language comprehension and expression, hand–eye coordination, social interaction, daily living activities and emotional regulation. The training environment is specially arranged, emphasising structured scheduling and visual prompts. Various methods such as verbal cues, written instructions, labels, body gestures and icons are employed to enhance the child’s understanding and mastery of the training content. This protocol was delivered in 30-minute sessions, six times weekly throughout the 12-week intervention period.

EBBS protocol: The therapeutic regimen incorporates the HB520D EEG biofeedback system as an adjunct to conventional rehabilitation. Following device initialisation, the main electrode output line is connected to crescent-shaped electrodes. The bilateral postauricular mastoid processes are cleansed with sterile saline and allowed to air-dry before electrode placement. The HB520D system delivers (Haobro, Suzhou, Jiangsu, China) transcranial alternating current stimulation at a frequency of 10 Hz (alpha band), with a biphasic square waveform (pulse width: 200 ms; inter-pulse interval: 50 ms). Current intensity is individualised, starting at 0.5 mA (35% device output) and titrated weekly by 0.1–0.2 mA based on real-time EEG feedback. Electrodes (Ag/AgCl, 5 cm^2^) are placed bilaterally over the mastoid processes (Cb1/Cb2 according to the 10–20 EEG system). Alpha Power Quantification: Spectral analysis was performed using fast Fourier transformation (FFT) to compute absolute alpha power (8~12 Hz) in the occipital regions. Real-time feedback was visualized via a dynamic spectrogram displaying alpha amplitude modulation. Target Achievement Criteria: Stimulation intensity (0.5~1.5 mA) was adjusted weekly based on the following thresholds: Baseline Phase: Achieve ≥70% alpha power enhancement in occipital regions compared to pre-intervention baseline for 5 consecutive minutes. Titration Phase: Increment current by 0.1~0.2 mA if alpha power remained below 85% of target; maintain or reduce intensity if ≥90% target was sustained. The EBBS protocol comprised daily 20-minute sessions, administered 5 days per week over a 12-week intervention period.

### Baseline Data Collection

Demographic and clinical characteristics including age, gender and disease duration were systematically recorded for all the participants.

### Detection of Cytokines

Serum biomarkers including 25-hydroxyvitamin D [25(OH)D], folic acid (FA) and brain-derived neurotrophic factor (BDNF) were measured at baseline and post intervention (within 24 hours after the final EBBS session). Blood samples were analysed using ELISA kits (manufacturer: BioVision Inc., Beijing, China) and processed on a BioTek Synergy H1 microplate reader (absorbance: 450 nm) following the manufacturer protocols. The intra- and inter-assay coefficients of variation were <8% and <12%, respectively.

### Scale Assessments

All the scales were administered at two time points: baseline (pre-intervention) and immediately post intervention (12 weeks). Assessments were conducted by trained clinicians blinded to group allocation.

(1) The Childhood autism rating scale (CARS) [15] assesses autism severity across 15 behavioural domains (e.g., social interaction and communication). It comprises 15 items scored on a 4-point Likert scale (1 = ‘normal for age’ to 4 = ‘severely abnormal’). The total score ranges from 15 to 60. Scores ≤29 indicate non-autistic, 30–36.5 indicate mild-to-moderate autism and ≥37 indicate severe autism. The Chinese version demonstrates Cronbach’s α = 0.92, test–retest reliability (ICC = 0.89) and convergent validity with ADOS-2 (r = 0.75) [16].

(2) The Montreal cognitive assessment (MoCA) [17] evaluates global cognitive function (attention, memory, language and visuospatial abilities). It comprises 30 items across seven domains. The total score ranges from 0 to 30. Scores ≥26 indicate normal cognition, and <26 suggest cognitive impairment. The Chinese version shows Cronbach’s α = 0.85, inter–rater reliability (κ = 0.82) and discriminant validity between ASD-ID and typical development (AUC = 0.91) [18].

(3) The developmental age is used to quantify developmental progress relative to chronological age and was calculated in this study using the Chinese psycho-educational profile. It evaluates five functional domains: gross motor, fine motor, language, adaptive behaviour and social skills. Each subdimension contains three items, each scored on a 0–2 scale, and the total score is converted to age-equivalent values. Linear interpolation can be performed if the total score spans two age stages (for example, the cognitive dimension score corresponds to 18–24 months and is calculated according to the actual score proportion). In terms of internal consistency, its Cronbach’s α = 0.89. Regarding convergent validity, it correlates with Bayley scales (r = 0.78) [19]. The developmental quotient (DQ) was calculated as [20] developmental age / chronological age × 100.

(4) The infants–junior middle school students’ social–life abilities scale (S–M) [21] examines six competency areas: selrhelp, locomorion, occupation, communication, socialization and self-direction. Result assessment: ≤5 is classified as extremely severe. A score of 6 is considered severe. 7 points moderate; 8 points is mild. 9 points is the edge. A score of 10 or above is normal. In terms of internal consistency, its Cronbach’s α = 0.88 (Chinese version). Regarding convergent validity, it is strongly correlated with the Vineland adaptive behaviour scale (r = 0.79) [22].

### Statistical Analysis

Statistical analysis was conducted using SPSS 26.0 software (IBM, Armonk, NY, USA). Categorical variables were compared using the chi-square test. For ordinal variables (e.g., ASD severity), non-parametric tests were performed (Mann–Whitney U test for two-group comparisons). Continuous variables first underwent normality assessment using the Shapiro–Wilk test to determine if the data conform to a normal distribution. Normally distributed data were analysed using independent samples *t*-tests and paired *t*-tests. Statistical significance was evaluated using the Benjamini-Hochberg (BH) procedure to control the false discovery rate (FDR) due to multiple comparisons. Significance was determined as follows: reject *H*_0_ if *p*_(𝑘)_ ≤ threshold_k_. Adjusted *p*-values (q-values) were computed as q_(𝑘)_ = min⋅[*p_(𝑘)_⋅*N/*k*,1]. All 16 tests remained significant after BH correction (q < 0.05). Adjusted *p*-values were reported for all outcomes, with the significance threshold set at *p* < 0.05.

## Results

### Comparison of Clinical Characteristics

The baseline characteristics (age, disease duration and gender) showed no statistically significant differences between the two groups (*p* > 0.05). Not only that, there were no differences in the course of ASD, family medical history, and severity between the two groups (*p* > 0.05), confirming their comparability (Table 1).

**Table 1.** **Clinical characteristics**.

		Conventional group (n = 163)	Observation group (n = 147)	Statistical	*p*
Age		4.25 ± 0.72	4.35 ± 0.81	t = 1.097	0.273
Boys		94 (57.67)	76 (51.70)	χ^2^ = 1.112	0.292
Girls		69 (42.33)	71 (48.30)		
Duration of ASD (months)		21.90 ± 5.69	22.39 ± 4.75	t = 0.812	0.418
Family history of ASD				χ^2^ = 0.467	0.494
	Yes	16 (9.82)	18 (12.24)		
	No	147 (90.18)	129 (87.76)		
Only child family				χ^2^ = 1.061	0.303
	Yes	152 (93.25)	141 (95.92)		
	No	11 (6.75)	6 (4.08)		
Degree of ASD				U = 3.000	0.700
	Mild	24 (14.72)	18 (12.24)		
	Moderate	94 (57.67)	93 (63.27)		
	Severe	45 (27.61)	36 (24.49)		

Note: ASD, Autism Spectrum Disorder.

### Comparison of ASD Improvement

No differences in baseline CARS score were observed between the two groups (*p* > 0.05). Both groups showed decreased CARS scores after intervention (*p* < 0.05). Compared with the conventional group, the observation group demonstrated significantly lower CARS scores after treatment (*p* < 0.05).

### Comparison of Serum Cytokine Levels

Baseline cytokine concentrations showed no intergroup differences for any measured biomarkers (*p* > 0.05). Post treatment, both groups exhibited significant elevation in 25(OH)D, FA and BDNF levels (*p* < 0.05). Particularly, the observation group showed significantly greater increases than the conventional group (*p* < 0.05).

### Comparison of Cognitive Function

Cognitive assessment demonstrated equivalent baseline performance on MoCA measures (*p* > 0.05). Following intervention, both groups showed cognitive enhancement (as evidenced by their increased MoCA scores). However, the observation group demonstrated significantly greater improvements than the conventional group (*p* < 0.05).

### Comparison of Developmental Age and DQ

Baseline DQ scores were similar between the groups (*p* > 0.05). Post treatment, the conventional group showed no significant changes in gross motor, adaptive behaviour or social skills (*p* > 0.05) but demonstrated improvement in fine motor and language domains (*p* < 0.05). The observation group exhibited significant improvement in all domains (gross motor, fine motor, language, adaptive behaviour and social skills), with scores significantly higher than those of the conventional group (*p* < 0.05).

### Comparison of Daily Living Skills

According to the S–M scale results, the conventional group showed improved scores in locomorion, socialization and self-direction (*p* < 0.05) but no significant changes in selrhelp, occupation or communication (*p* > 0.05). By contrast, the observation group demonstrated significant improvement in all S–M domains, with scores significantly higher than those of the conventional group (*p* < 0.05).

### Adjustment of Results

Using the Benjamini-Hochberg procedure, all 16 post-hoc tests retained statistical significance after adjusting for multiple comparisons (adjusted q-values <0.05; see Tables 2,3,4,5,6 for detailed results).

**Table 2.** **ASD improvement**.

		Conventional group (n = 163)	Observation group (n = 147)	t	*p* (raw)	*k*	BH Threshold (α*·k/16*)	*p* (adjusted)
CARS	Baseline	37.94 ± 6.16	38.75 ± 7.12	1.074	0.284	-	-	-
	After treatment	31.61 ± 5.04	28.95 ± 5.05	4.651	<0.001	11	0.034	0.001
	t	32.973	13.621					
	*p*	<0.001	<0.001					

Note: CARS, Childhood Autism Rating Scale; BH, Benjamini-Hochberg.

**Table 3.** **Serum cytokine levels**.

		Conventional group (n = 163)	Observation group (n = 147)	t	*p* (raw)	*k*	BH Threshold (α*·k/16*)	*p* (adjusted)
25(OH)D (ng/mL)	Baseline	24.68 ± 3.70	24.38 ± 4.97	0.595	0.553	-	-	-
	After treatment	28.51 ± 5.16	31.37 ± 5.66	4.664	<0.001	4	0.013	0.001
	t	7.699	11.251					
	*p*	<0.001	<0.001					
FA (ng/mL)	Baseline	15.39 ± 3.35	15.57 ± 4.16	0.428	0.669	-	-	-
	After treatment	20.66 ± 4.08	22.85 ± 4.18	4.674	<0.001	3	0.009	0.001
	t	12.731	14.968					
	*p*	<0.001	<0.001					
BDNF (ng/mL)	Baseline	5.24 ± 1.08	5.13 ± 1.29	0.834	0.405	-	-	-
	After treatment	6.41 ± 1.53	7.20 ± 1.59	4.458	<0.001	2	0.006	0.001
	t	7.987	12.313					
	*p*	<0.001	<0.001					

Note: 25(OH)D, 25-Hydroxyvitamin D; FA, Folic Acid; BDNF, Brain-Derived Neurotrophic Factor; BH, Benjamini-Hochberg.

**Table 4.** **Cognitive function**.

		Conventional group (n = 163)	Observation group (n = 147)	t	*p* (raw)	*k*	BH Threshold (α*·k/16*)	*p* (adjusted)
MoCA	Baseline	16.99 ± 3.70	17.22 ± 3.27	0.578	0.563	-	-	-
	After treatment	23.32 ± 4.05	24.76 ± 4.27	3.039	0.003	13	0.041	0.003
	t	14.243	16.973					
	*p*	<0.001	<0.001					

Note: MoCA, Montreal Cognitive Assessment; BH, Benjamini-Hochberg.

**Table 5.** **Developmental age and DQ**.

		Conventional group (n = 163)	Observation group (n = 147)	t	*p* (raw)	*k*	BH Threshold (α*·k/16*)	*p* (adjusted)
Gross motor	Baseline	64.76 ± 10.40	64.15 ± 9.58	0.536	0.592	-	-	-
	After treatment	66.19 ± 9.70	78.00 ± 8.32	11.443	<0.001	1	0.003	0.001
	t	1.283	13.241					
	*p*	0.200	<0.001					
Fine motor	Baseline	51.07 ± 7.33	52.42 ± 7.05	1.646	0.101	-	-	-
	After treatment	62.18 ± 8.51	65.20 ± 9.29	2.980	0.003	14	0.044	0.003
	t	12.631	13.281					
	*p*	<0.001	<0.001					
Language	Baseline	41.98 ± 9.26	41.29 ± 8.36	0.685	0.494	-	-	-
	After treatment	51.50 ± 7.55	54.34 ± 8.50	3.119	0.002	12	0.038	0.003
	t	10.174	13.274					
	*p*	<0.001	<0.001					
Adaptive behaviour	Baseline	49.79 ± 8.47	49.35 ± 7.70	0.482	0.631	-	-	-
	After treatment	49.81 ± 8.42	53.50 ± 7.50	4.053	<0.001	5	0.016	0.001
	t	0.020	4.678					
	*p*	0.984	<0.001					
Social skills	Baseline	48.66 ± 9.57	49.36 ± 7.92	0.695	0.487	-	-	-
	After treatment	49.99 ± 8.91	52.72 ± 6.59	3.045	0.003	15	0.047	0.003
	t	1.294	3.955					
	*p*	0.200	<0.001					

Note: DQ, Developmental Quotient; BH, Benjamini-Hochberg.

**Table 6.** **S–M scores**.

		Conventional group (n = 163)	Observation group (n = 147)	t	*p* (raw)	*k*	BH Threshold (α*·k/16*)	*p* (adjusted)
Selrhelp	Baseline	6.71 ± 3.28	6.14 ± 2.36	1.736	0.084	-	-	-
	After treatment	6.96 ± 1.82	7.52 ± 1.87	2.639	0.009	16	0.050	0.009
	t	0.876	5.552					
	*p*	0.382	<0.001					
Locomorion	Baseline	3.58 ± 1.14	3.63 ± 1.43	0.294	0.769	-	-	-
	After treatment	4.12 ± 1.21	4.92 ± 1.14	5.995	<0.001	6	0.019	0.001
	t	4.105	8.559					
	*p*	<0.001	<0.001					
Occupation	Baseline	3.93 ± 1.26	4.01 ± 1.28	0.333	0.739	-	-	-
	After treatment	4.06 ± 1.24	4.98 ± 1.10	8.123	<0.001	7	0.022	0.001
	t	1.361	6.931					
	*p*	0.175	<0.001					
Communication	Baseline	3.66 ± 1.34	3.64 ± 1.47	0.106	0.916	-	-	-
	After treatment	3.67 ± 1.45	4.22 ± 1.38	3.445	<0.001	8	0.025	0.001
	t	0.079	3.495					
	*p*	0.937	<0.001					
Socialization	Baseline	3.46 ± 1.37	3.45 ± 1.41	0.071	0.944	-	-	-
	After treatment	4.53 ± 1.30	5.16 ± 1.32	4.215	<0.001	9	0.028	0.001
	t	7.221	10.734					
	*p*	<0.001	<0.001					
Self-direction	Baseline	1.63 ± 0.52	1.73 ± 0.45	1.733	0.084	-	-	-
	After treatment	2.17 ± 0.71	2.67 ± 0.65	6.556	<0.001	10	0.031	0.001
	t	7.775	7.775					
	*p*	<0.001	<0.001					

Note: S–M, Infants–Junior Middle School Students’ Social–Life Abilities Scale; BH, Benjamini-Hochberg.

## Discussion

Different from conventional therapies that primarily target behavioural modification through external reinforcement [23], EBBS directly modulates neural activity via bioelectric stimulation [24]. This dual-action mechanism—normalising aberrant EEG oscillations while enhancing synaptic plasticity—enables simultaneous improvements in cognitive function (e.g., MoCA scores) and behavioural symptoms (e.g., CARS reduction). Such multidimensional efficacy is critical for ASD–ID comorbidity, where ID-related cognitive impairments often hinder responsiveness to behavioural training alone.

Comparative analysis of ASD symptom improvement revealed superior outcomes in the observation group, as evidenced by their significantly lower CARS scores compared with those of the controls. Current clinical research has confirmed that conventional comprehensive rehabilitation therapy works by guiding children with ASD to participate in various activities, thereby training and enhancing their neuronal responsiveness. As a core component of this approach, ABA specifically aims to modify abnormal behaviours in ASD while promoting the development of multiple competencies. Meanwhile, structured teaching capitalises on visual learning strengths to enhance environmental perception and task execution while reducing anxiety and stress when confronted with unfamiliar situations [25]. Nevertheless, for children with ASD–ID comorbidity, the cognitive and developmental limitations characteristic of ID markedly impair their learning capacity and training responsiveness, resulting in their suboptimal responses to conventional rehabilitation strategies [26].

In this study, the superior ASD improvement observed in the observation group may be mechanistically explained by several factors: (1) EBBS operates through the cerebellum–thalamus–cerebral cortex neural network, with direct projections to the cerebellar fastigial nucleus via cortical pathways. This dual mechanism of cortical excitation coupled with neurodevelopmental stimulation significantly contributes to cerebral functional enhancement [27]. The significantly elevated post-treatment levels of 25(OH)D [28] and FA [29] in the observation group relative to those of the controls can also confirm our view. The observed elevation in 25(OH)D and FA may reflect the secondary effects of EBBS. Enhanced neural activity and cerebral perfusion can improve nutrient absorption or metabolic regulation. Alternatively, behavioural improvements (e.g., reduced food selectivity) might indirectly increase dietary intake of vitamin D and folate. However, the direct causal links require further investigation. (2) Axonal shortening, degeneration and transport impairment are characteristic neuropathological features in ID [30]. Experimental evidence from Athavale *et al*. [31] animal studies indicates that EBBS exhibits potent axonal regeneration-inducing properties that facilitate synaptic remodelling. Such effects benefit the recovery of cognitive learning and memory processes. The significant post-treatment elevation of BDNF levels in the observation group provides further confirmation, as BDNF is known to mediate neuronal regeneration, differentiation and developmental processes and play a vital role in the repair, injury and development of the nervous system [32]. These findings are corroborated by Villalobos J *et al*.’s [33] demonstration of EBBS-mediated neurological improvement in diabetic rat models, showing remarkable consistency with our clinical observations. (3) EBBS employs advanced digital frequency synthesis technology to transform specific pulse sequences and bioelectrical signals into EEG-simulated bio-currents. These precisely modulated currents are then delivered through bilateral mastoid (postauricular) electrodes to target the cerebellar fastigial nucleus region. Leveraging the principles of fastigial nucleus electrical stimulation, this innovative approach induces beneficial neuroplastic changes, including brain tissue reorganisation and enhanced cerebral blood perfusion [34]. Our study results provide compelling evidence for EBBS’s cognitive-enhancing effects, with the observation group demonstrating significantly superior post-treatment MoCA scores compared with the control participants.

Longitudinal assessment revealed pronounced improvements in developmental age and DQ among the EBBS-treated subjects. As a standardised metric for evaluating intellectual functioning in children with intellectual disabilities, developmental age and DQ calculation involves the ratio of mental age to chronological age. During the treatment course, the developmental age and DQ can register a positive elevation only when the increment in mental age surpasses that of the chronological age [35]. The observed increase in developmental age and DQ indicates that the mental age progression in the observation group outpaced normal chronological aging during the treatment period, representing meaningful cognitive gains. The treatment response rate in this study directly reflects the intervention efficacy within the group, corroborating the effectiveness of the EBBS protocol. These findings align with those reported by Wang C *et al*. [36], who investigated the application of EBBS in treating refractory hypertension. Quality of life assessments using the S–M scale further corroborated these positive outcomes, with the observation group showing greater improvements in daily functioning and overall quality of life compared with the controls. An interesting methodological observation revealed apparent discrepancies between fine motor skill assessments (developmental age and DQ) and functional ability measures (S–M scale). This discordance reflects fundamental differences in the assessment’s focus rather than contradictory results—the developmental age and DQ demands highly precise fine-finger movements from children, whereas the S–M scale places emphasis on the functional operations and capabilities accomplished by children using both hands. Nevertheless, the research group acknowledge the possibility of random variation influencing these outcomes, necessitating further investigation through large-scale studies.

For ASD–ID intervention, EBBS should be integrated as an adjunct to multidisciplinary frameworks. For instance, combining EBBS with ABA therapy could synergise neuromodulation (targeting cognitive deficits) and behavioural shaping (addressing social communication). Future protocols might sequence EBBS sessions before structured teaching to prime neural responsiveness. Additionally, serum biomarker (e.g., BDNF) monitoring could personalise stimulation parameters, ensuring optimal synergy with pharmacological or occupational therapies. Such integration requires collaborative efforts among neurologists, psychologists and rehabilitation specialists to tailor multimodal interventions.

As a retrospective study, this research lacks a sham stimulation control group. Although the observation group showed significant improvements compared with the control group, the placebo effect cannot be entirely ruled out. Future randomised controlled trials should incorporate blinded sham-controlled designs to isolate the specific effects of EBBS. Additionally, the absence of supporting *in vitro* studies prevents us from drawing definitive conclusions about EBBS’s precise biological mechanisms, representing an important area for subsequent investigation.

## Conclusion

This study provides substantive evidence that EBBS intervention significantly improves cognitive function, behavioural symptoms and adaptive living skills among children with comorbid ASD–ID. The parallel elevations observed in serum 25(OH)D, FA and BDNF levels suggest that EBBS’s therapeutic effects may be mediated through the dual mechanisms of neural circuit remodelling and metabolic pathway modulation.

## Availability of Data and Materials

The data used and/or analyzed during the current study are available from the corresponding author.

## References

[1] Hirota T, King BH (2023). Autism Spectrum Disorder: A Review. *JAMA*.

[2] Maguire E, Mulryan N, Sheerin F, McCallion P, McCarron M (2022). Autism spectrum disorder in older adults with intellectual disability: a scoping review. *Irish Journal of Psychological Medicine*.

[3] Stefanski A, Calle-López Y, Leu C, Pérez-Palma E, Pestana-Knight E, Lal D (2021). Clinical sequencing yield in epilepsy, autism spectrum disorder, and intellectual disability: A systematic review and meta-analysis. *Epilepsia*.

[4] Xie S, Karlsson H, Dalman C, Widman L, Rai D, Gardner RM (2020). The Familial Risk of Autism Spectrum Disorder with and without Intellectual Disability. *Autism Research: Official Journal of the International Society for Autism Research*.

[5] Marrus N, Koth KA, Hellings JA, McDonald R, Gwynette MF, Muhle R (2023). Psychiatry training in autism spectrum disorder and intellectual disability: Ongoing gaps and emerging opportunities. *Autism: the International Journal of Research and Practice*.

[6] Kayarian FB, Jannati A, Rotenberg A, Santarnecchi E (2020). Targeting Gamma-Related Pathophysiology in Autism Spectrum Disorder Using Transcranial Electrical Stimulation: Opportunities and Challenges. *Autism Research: Official Journal of the International Society for Autism Research*.

[7] Casanova MF, Shaban M, Ghazal M, El-Baz AS, Casanova EL, Sokhadze EM (2021). Ringing Decay of Gamma Oscillations and Transcranial Magnetic Stimulation Therapy in Autism Spectrum Disorder. *Applied Psychophysiology and Biofeedback*.

[8] Schlaeppi JA, Affentranger L, Bervini D, Z’Graggen WJ, Raabe A, Pollo C (2022). Electrical Stimulation for Cerebral Vasospasm After Subarachnoid Hemorrhage: A Systematic Review. *Neuromodulation: Journal of the International Neuromodulation Society*.

[9] Krauel K, Brauer H, Breitling-Ziegler C, Freitag CM, Luckhardt C, Mühlherr A (2025). Prefrontal Transcranial Direct Current Stimulation in Pediatric Attention-Deficit/Hyperactivity Disorder: A Randomized Clinical Trial. *JAMA Network Open*.

[10] Azimi S, Lima F, Slack-Smith L, Bourke J, Calache H, Junaid M (2022). Factors associated with dental hospitalisations in children with intellectual disability or autism spectrum disorder: a Western Australian population-based retrospective cohort study. *Disability and Rehabilitation*.

[11] Rosello R, Martinez-Raga J, Mira A, Girela B, Cortese S (2021). Developmental outcomes in adolescence of children with autism spectrum disorder without intellectual disability: A systematic review of prospective studies. *Neuroscience and Biobehavioral Reviews*.

[12] Prillinger K, Amador de Lara G, Klöbl M, Lanzenberger R, Plener PL, Poustka L (2024). Multisession tDCS combined with intrastimulation training improves emotion recognition in adolescents with autism spectrum disorder. *Neurotherapeutics: the Journal of the American Society for Experimental NeuroTherapeutics*.

[13] Idema WJ, Konz DN, Mulder AF, Kattentidt-Mouravieva AA, Kasius MC, Ester WA (2022). Care for adolescents with an autism spectrum disorder and an intellectual disability. *Tijdschrift voor Psychiatrie*.

[14] First MB (2013). Diagnostic and statistical manual of mental disorders, 5th edition, and clinical utility. *The Journal of Nervous and Mental Disease*.

[15] Schopler E, Reichler RJ, DeVellis RF, Daly K (1980). Toward objective classification of childhood autism: Childhood Autism Rating Scale (CARS). *Journal of Autism and Developmental Disorders*.

[16] Chu JH, Bian F, Yan RY, Li YL, Cui YH, Li Y (2022). Comparison of diagnostic validity of two autism rating scales for suspected autism in a large Chinese sample. *World Journal of Clinical Cases*.

[17] Nasreddine ZS, Phillips NA, Bédirian V, Charbonneau S, Whitehead V, Collin I (2005). The Montreal Cognitive Assessment, MoCA: a brief screening tool for mild cognitive impairment. *Journal of the American Geriatrics Society*.

[18] Chen KL, Xu Y, Chu AQ, Ding D, Liang XN, Nasreddine ZS (2016). Validation of the Chinese Version of Montreal Cognitive Assessment Basic for Screening Mild Cognitive Impairment. *Journal of the American Geriatrics Society*.

[19] Shek DTL, Tsang SKM, Lam LL, Tang FLY, Cheung PMP (2005). Psychometric properties of the Chinese version of the Psycho-educational Profile-Revised (CPEP-R). *Journal of Autism and Developmental Disorders*.

[20] Harel-Gadassi A, Friedlander E, Yaari M, Bar-Oz B, Eventov-Friedman S, Mankuta D (2018). Developmental assessment of preterm infants: Chronological or corrected age?. *Research in Developmental Disabilities*.

[21] Geng J, Lou T, Liu PP, Gou L, Sun AM (2024). Therapeutic effectiveness of bionic electrical stimulation combined with conventional rehabilitation for mild and moderate intellectual disability in children. *Chinese Journal of Rehabilitation*.

[22] Mao SJ, Shen J, Xu F, Zou CC (2019). Quality of life in caregivers of young children with Prader-Willi syndrome. *World Journal of Pediatrics: WJP*.

[23] Gray HL, Pang T, Agazzi H, Shaffer-Hudkins E, Kim E, Miltenberger RG (2022). A nutrition education intervention to improve eating behaviors of children with autism spectrum disorder: Study protocol for a pilot randomized controlled trial. *Contemporary Clinical Trials*.

[24] Wang M, Wang T, Li X, Yuan Y (2023). Low-intensity ultrasound stimulation modulates cortical neurovascular coupling in an attention deficit hyperactivity disorder rat model. *Cerebral Cortex (New York, N.Y.: 1991)*.

[25] Çıkrıkçı Ö, Çıkrıkçı N, Griffiths M (2022). Fear of COVID-19, stress and depression: A meta-analytic test of the mediating role of anxiety. *Psychol Psychother*.

[26] Culnane E, Efron D, Williams K, Marraffa C, Antolovich G, Prakash C (2023). Carer perspectives of a transition to adult care model for adolescents with an intellectual disability and/or autism spectrum disorder with mental health comorbidities. *Child: Care, Health and Development*.

[27] Jannati A, Oberman LM, Rotenberg A, Pascual-Leone A (2023). Assessing the mechanisms of brain plasticity by transcranial magnetic stimulation. *Neuropsychopharmacology: Official Publication of the American College of Neuropsychopharmacology*.

[28] Petruzzelli MG, Marzulli L, Margari F, De Giacomo A, Gabellone A, Giannico OV (2020). Vitamin D Deficiency in Autism Spectrum Disorder: A Cross-Sectional Study. *Disease Markers*.

[29] Liu X, Zou M, Sun C, Wu L, Chen WX (2022). Prenatal Folic Acid Supplements and Offspring’s Autism Spectrum Disorder: A Meta-analysis and Meta-regression. *Journal of Autism and Developmental Disorders*.

[30] Hu W, Zou L, Yu N, Wu Z, Yang W, Wu T (2024). Catalpol rescues LPS-induced cognitive impairment via inhibition of NF-Κb-regulated neuroinflammation and up-regulation of TrkB-mediated BDNF secretion in mice. *Journal of Ethnopharmacology*.

[31] Athavale ON, Cheng LK, Avci R, Clark AR, Du P (2023). Cervical Vagus Nerve Stimulation Disrupts Gastric Slow Wave Activity in Rats. *Annual International Conference of the IEEE Engineering in Medicine and Biology Society. IEEE Engineering in Medicine and Biology Society. Annual International Conference*.

[32] Chen X, Chen A, Wei J, Huang Y, Deng J, Chen P (2024). Dexmedetomidine alleviates cognitive impairment by promoting hippocampal neurogenesis via BDNF/TrkB/CREB signaling pathway in hypoxic-ischemic neonatal rats. *CNS Neuroscience & Therapeutics*.

[33] Villalobos J, Payne SC, Ward GM, Andrikopoulos S, Hyakumura T, MacIsaac RJ (2023). Stimulation parameters for directional vagus nerve stimulation. *Bioelectronic Medicine*.

[34] Rinaldi A, Martins MCM, Maioli M, Rinaldi S, Fontani V (2023). REAC Noninvasive Neurobiological Stimulation in Autism Spectrum Disorder for Alleviating Stress Impact. *Advances in Neurodevelopmental Disorders*.

[35] Maeda T, Tanahashi Y, Asada H, Kidokoro H, Takahashi Y, Sato Y (2024). High threshold of total developmental quotient at 3 years for follow-up in extremely preterm infants. *Early Human Development*.

[36] Wang C, Wang P, Qi G (2023). A new use of transcutaneous electrical nerve stimulation: Role of bioelectric technology in resistant hypertension (Review). *Biomedical Reports*.

